# Ternary Phase-Separation Investigation of Sol-Gel Derived Silica from Ethyl Silicate 40

**DOI:** 10.1038/srep14560

**Published:** 2015-09-28

**Authors:** Shengnan Wang, David K. Wang, Simon Smart, João C. Diniz da Costa

**Affiliations:** 1 The University of Queensland, FIM^2^Lab – Functional Interfacial Materials and Membranes Laboratory, School of Chemical Engineering, Brisbane Qld 4072, Australia

## Abstract

A ternary phase-separation investigation of the ethyl silicate 40 (ES40) sol-gel process was conducted using ethanol and water as the solvent and hydrolysing agent, respectively. This oligomeric silica precursor underwent various degrees of phase separation behaviour in solution during the sol-gel reactions as a function of temperature and H_2_O/Si ratios. The solution composition within the immiscible region of the ES40 phase-separated system shows that the hydrolysis and condensation reactions decreased with decreasing reaction temperature. A mesoporous structure was obtained at low temperature due to weak drying forces from slow solvent evaporation on one hand and formation of unreacted ES40 cages in the other, which reduced network shrinkage and produced larger pores. This was attributed to the concentration of the reactive sites around the phase-separated interface, which enhanced the condensation and crosslinking. Contrary to dense silica structures obtained from sol-gel reactions in the miscible region, higher microporosity was produced via a phase-separated sol-gel system by using high H_2_O/Si ratios. This tailoring process facilitated further condensation reactions and crosslinking of silica chains, which coupled with stiffening of the network, made it more resistant to compression and densification.

Silica porous materials have attracted growing scientific interest due to their unique properties in terms of large surface area, thermal stability and chemical inertness and consequently have found diverse applications in absorption^1^, catalysis^2^, energy^2^ and separation applications^3^. The silica sol-gel method generally comprises of reacting a silica precursor in the presence of solvents and catalysts by the well-established hydrolysis and condensation reactions. Here, the reactant ratios, pH of the solution, reaction temperature, and nature of silica precursor all affect the reaction mechanisms and kinetics^4^,^5^ and the final xerogel structure. To effectively tailor the porosity of xerogels by the sol-gel method, it is necessary to understand how the reactions influence the porous structure formation, arising from the formation of silanol (Si-OH) groups via hydrolysis and siloxane (Si-O-Si) bridges via the condensation reaction.

The use of Fourier transform infrared spectroscopy (FTIR) is widely used for examining the evolution of the silica frameworks through their functional groups in a sol-gel reaction system^6^,^7^,^8^,^9^, and xerogel characterisation^10^,^11^,^12^. However, FTIR analysis of aqueous silica sol-gel is seldom reported. In a few examples, Tejedor-Tejedor *et al.* monitored the hydrolysis and condensation reactions of tetraethyl orthosilicate (TEOS) under rich water conditions and suggested that the hydrolysis is a first-order reaction^13^. In another work, Jiang *et al.* investigated the activation energy and Arrhenius factor of the hydrolysis of methyltriethoxysilane under different temperatures^14^. Further, Neville *et al.* followed the sol-gel process of methyltrimethoxysilane by measuring the peak intensity variation of silanol (Si-OH) groups generated from hydrolysis and siloxane bridges (Si-O-Si) from condensation and in so doing, introduced the silica particle growth mechanism^15^. These studies strongly suggest that FTIR is a strong characterisation tool for assessing the silica sol-gel process.

ES40 is a partially-condensed form of TEOS, with five silicon atoms per molecule on average, thus providing higher silica content but lower solubility in aqueous solutions. ES40 became more attractive in recent years due to higher silica productivity thus making this silica precursor economically desirable for a range of applications. Of particular attention, ES40 xerogels delivered superior thermal stability than analogous TEOS xerogels^16^. Recently, Wang *et al.* produced ES40-derived silica/cobalt membranes by rapid thermal processing techniques which showed superior performance as membrane films that otherwise could not be achieved with TEOS^17^. ES40 has also been found to improve the hydrothermal stability of silica when prepared at high water and low ethanol contents^18^. Considering these desirable aspects, it is important to study the ES40 sol-gel process in order to better tailor materials.

In principle, the preparation of homogeneous solutions is preferable when using the silica sol-gel method, particularly when it involves thin film coating and/or controlling the porous structure. However, in this work we show that, under our testing conditions, ES40 tends to form a heterogeneous two phase system induced by phase separation behaviour. Therefore, this work investigates the phase-separated sol-gel process leading to the formation of porous silica, in contrast to the reported work on homogeneous ES40 sol-gel leading to extremely microporous or ultimately dense silica. The evolution of the phase-separated ES40 sol-gel method is studied as a function of reaction temperatures and molar ratios of water to ES40 (H_2_O/ES40).

## Results and Discussion

**Figure 1:**
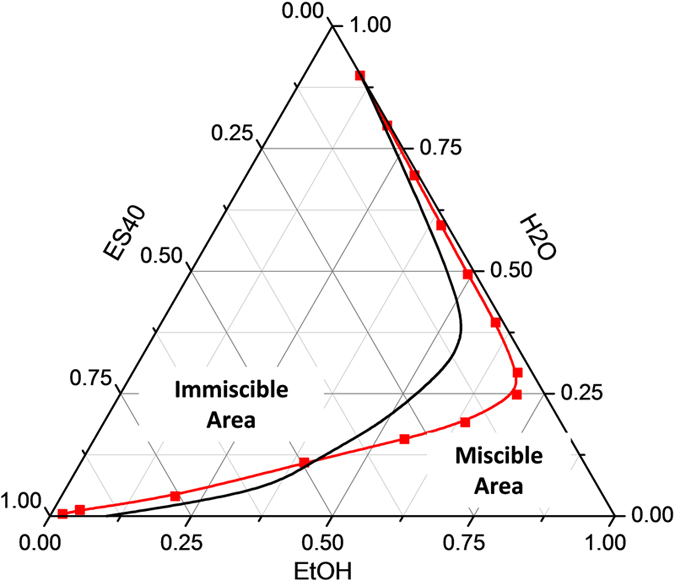
Ternary phase diagrams of ES40-ethanol- water (red line) and TEOS-ethanol-water system (black line) at 25 °C.

Figure 1 shows the ternary phase diagram of an ES40-ethanol-water system. The red miscibility line which divides the diagram into miscible and immiscible regions was determined by visual inspection of the miscibility of mixtures. The black boundary line for TEOS is adapted from Brinker and Scherer without modification^4^. It is evident that ES40 exhibited a lower solubility than TEOS in water-ethanol mixtures as seen by the reduced miscible area in Fig. 1. Such behaviour is manifested by its longer molecular chains of the precursor, as well as the ability to form larger silica particles during the hydrolysis and condensation sol-gel process^17^,^19^. Due to these factors, the extent of phase separation of the growing silica species is heightened, and should be carefully monitored.

**Figure 2:**
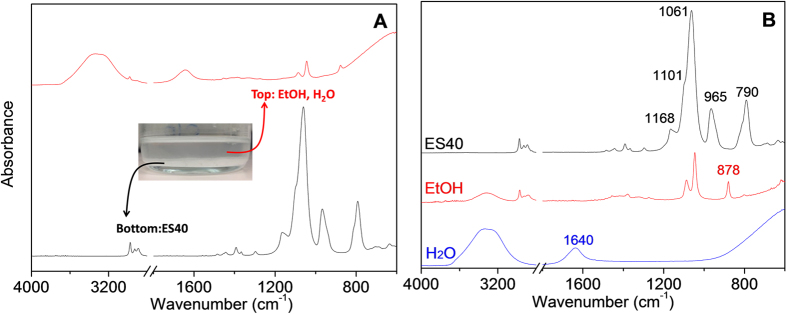
FTIR spectra of (A) sol-gel solutions before drying with photo (inset) and (B) pure ES40, ethanol and water.

The phase-separated sol-gel of ES40 in acidic ethanol-water solutions was characterised by ATR-FTIR. Phase separation was obvious from the beginning of the sol-gel process as shown by the inset photo in Fig. 2A. The FTIR spectrum (at time = 0) of the cloudy phase on the bottom as indicated on Fig. 2A is identical with that of pure ES40 (Fig. 2B), while the clear solution on the top is a mixture of only water and ethanol species.

Table 1 summarizes the correlations between the frequencies and vibration modes based on the literature^20^,^21^,^22^,^23^. The broad peak observed at around 3320 cm^−1^ is attributed to the O-H stretching vibration of H_2_O and EtOH. The appearance of this peak is affected by environment, including the neighbouring network and/or hydrogen bonds connected to O-H. The weak bands located in the region of 3000–2800 cm^−1^ are assigned to C-H stretching vibrations of ethanol and Si-OCH_2_CH_3_. A H-O-H deformation band appears exclusively at 1640 cm^−1^ in the pure H_2_O spectrum, which was used to monitor water in the samples. The peak at 878 cm^−1^ is the characteristic absorption of EtOH, which is assigned to C-C and C-O stretching vibrations. The C-O stretching of the silica precursor is associated with the absorption band at 790 cm^−1^. Numerous absorption bands appear between 1200 cm^−1^ to 900 cm^−1^. Besides the C-O/C-C stretching vibration (1086 cm^−1^) and CH_3_/CH_2_ rocking (1045 cm^−1^) of EtOH, CH_3_ rocking (1168 and 965 cm^−1^) and C-O asymmetric stretching (1101 and 1061 cm^−1^) of silica precursor also exhibit within this range.

**Figure 3:**
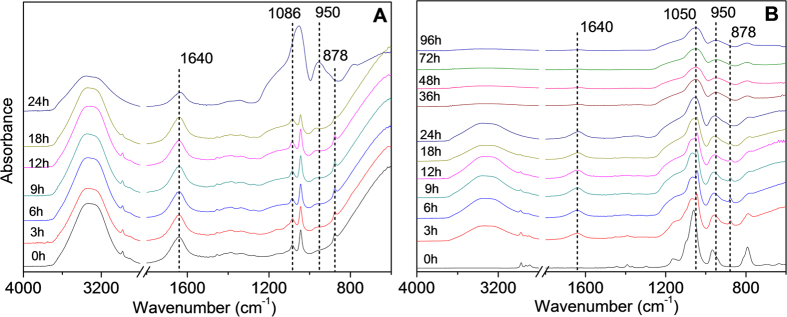
FTIR spectra of the (A) top phase and (B) bottom phase of ES40 sol-gel solutions at different drying times.

Figure 3 shows the FTIR spectra of the phase separated sol-gel system at different time intervals during the gelling stage of samples drying at 60 °C. The appearance of H-O-H deformation band at 1640 cm^−1^ in the top phase (Fig. 3A) and intermittently from 6 to 24 h in the bottom phase (Fig. 3B) confirms the existence of water, which could be attributed to the co-solvent or a condensation by-product. After 24 h of gelling, the sol mixture was no longer liquid and only the bottom phase remained as a hard gel. This is consistent with the gradual decreasing C-H stretching vibrations of ethanol and water around 3000–2800 cm^−1^ due to the evaporation of solvents and the by-products of ethanol and water arising from hydrolysis and condensation reactions. This is supported by the characteristic absorption of EtOH at 878 cm^−1^, as a single peak, which also decreased intensity, corresponding with ethanol evaporation. Therefore, it can be concluded that the presence of ethanol and water species were insignificant after the initial 24 h of gelation during drying.

Another important observation to be made from Fig. 3 is that most of the absorption bands of silicon-containing sol-gel derived materials are located in the region of 1200 to 900 cm^−1^. The frequency of CH_3_ rocking in ES40 alkoxy groups (~965 cm^−1^) shifted to lower wavenumber (~950 cm^−1^) in Fig. 3B and is attributed to the replacement of Si-OCH_2_CH_3_ by Si-OH. The intensity of silanol groups at ~950 cm^−1^ increases with reaction time. The shape of this band became wider and increasingly asymmetric, implying that this band should include two constituents as reported elsewhere; one at ~960 cm^−1^ corresponding to silanol and the other one at ~930 cm^−1^ attributed to the deprotonated form (Si-O^−^)^24^. In contrast, the evolution of siloxane bands is less straight-forward in comparison to the silanol bands as it is overlapped by the vibration peaks attributing to ethanol between 1200 and 1000 cm^−1^ as shown in Fig. 2B. However, it is noticeable that the intensity ratio of the 1085 cm^−1^ peak to the 1045 cm^−1^ peak is not equal to that in the pure ethanol. This ratio increases over time, which is exclusively associated to the more intense absorption of the siloxane groups due to the on-going condensation reactions. These observations agree well with earlier reports on silica sol-gel process^15^,^25^,^26^,^27^.

These results indicate that ES40 sol-gel reaction did occur in the heterogeneous phase-separated systems. It is hypothesized that hydrolysis took place at the interface of the two phases. As the silica polymerization progressed, the ethoxyl groups bonded to the silica atoms (Si-OEt) in the bottom phase turned into hydrophilic silanol groups (Si-OH), which subsequently produced the siloxane groups (Si-O-Si) as evidenced by the increasing intensity of the 950 cm^−1^ and 1065 cm^−1^ peaks. After further condensation and solvent evaporation (ethanol and water), the sol-gel solution became a single phase and eventually formed xerogels after drying.

**Figure 4:**
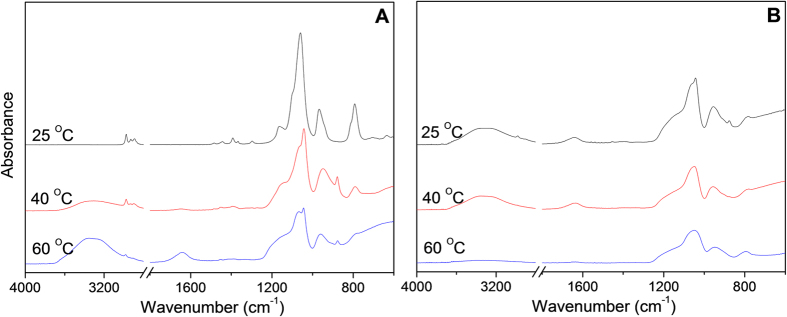
FTIR spectra of the bottom phase of ES40 sol-gel solutions after drying for (A) 9 h and (B) 72 h at 25, 40 and 60 °C.

Besides the investigation of sol-gel process at 60 °C, different temperatures during the gelling stage were also investigated and analysed using the same FTIR methodology. The evolution of the silica structure at 25, 40 and 60 °C at 9 and 72 h of drying time in the bottom phase are shown Fig. 4 (temporal evolution of full FTIR spectra for the 25 and 40 °C samples are supplied in ESI). As shown in Fig. 4A, the spectrum only shows the pure ES40 absorption peaks at 25 °C, indicating no detectable reaction has taken place after 9 h, while the extent of hydrolysis at 40 and 60 °C is much greater as manifested by the reduced C-O vibration at ~780 cm^−1^ and the C-H stretching vibrations of Si-OCH_2_CH_3_ at 2800 cm^−1^, which almost vanishes after 9 h at 60 °C. In addition, the degree of condensation is furthered at higher temperature conditions as demonstrated by the broadening of the absorption peak at ~1150 cm^−1^ (Si-O-Si). At 72 h, as seen in Fig. 4B, the condensation of silica at different drying temperatures is also different. The absorbance intensity of uncondensed silica species, Si-OH and Si-O^−^, at ~950 cm^−1^ is much weaker at 60 °C system compared to that in the 40 and 25 °C spectra. These results demonstrate that the sol-gel process in phase-separated system is significantly affected by reaction temperature in this study.

**Figure 5:**
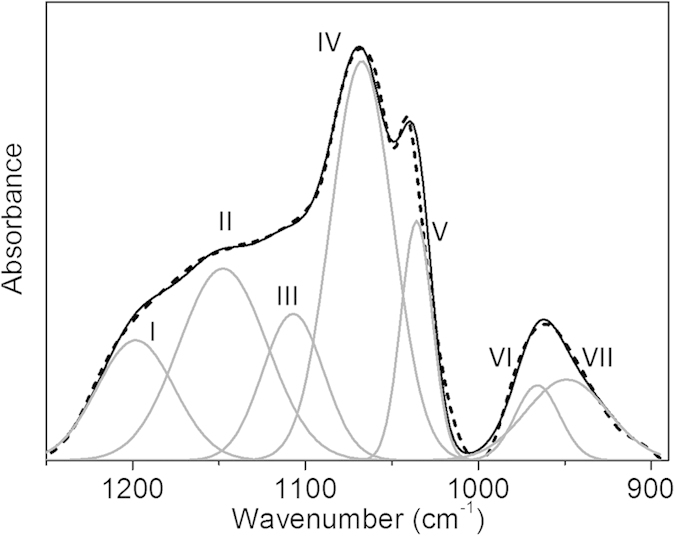
FTIR spectrum (dotted line) and peak deconvolution of the bottom phase for 9 h sample dried at 60 °C after EtOH spectral subtraction. The solid lines are summation (black) of the fitted peaks (grey) with an R^2^ value ≥ 0.995.

Further analysis of the FTIR spectra provided meaningful information about the subtle differences in these phase-separated sol-gel systems by measuring the intensity of absorption corresponding to the various chemical species, i.e. Si-OH and Si-O-Si. In silica sol-gel process, the quantification of absorption peaks relating to silanol and siloxane species could provide some valuable insight into the extent of hydrolysis and condensation in the sol-gel process. However, due to the overlapping nature between the absorption peaks of ethanol and ES40 in the region of between 1200 and 1000 cm^−1^, careful spectral subtraction was carried out to remove the contribution of the ethanol solvent in the initial sol-gel reaction (for times <24 h) according to Tejedor-Tejedor *et al.*^13^. To perform a quantitative analysis, deconvolution of the peaks was used to identify various vibrations in the overlapping regions. Figure 5 illustrates a representative example of peak deconvolution of a spectrum (60 °C, 9 h) after ethanol spectral subtraction and the assignment of the fitted peaks is summarized in Table 2.

As shown in Fig. 5, peaks VI and VII are corresponding to the products of the hydrolysis reaction for Si-OH and Si-O^−^ bonds, respectively. While for the broad band in the region 1250 to 1000 cm^−1^, five fitted peaks centred at ~1205, 1146, 1105, 1065 and 1035 cm^−1^ are related to product of condensation reaction Si-O-Si bond. According to the literature, the absorption at peak V (1035 cm^−1^) is characteristic of chain and sheet silicates^13^ The peaks II (1146 cm^−1^) and III (1105 cm^−1^) have been assigned to the longitudinal optical (LO)-transverse optical (TO) splitting modes of 4-ring siloxane, while the other two absorption I (1205 cm^−1^) and IV (1065 cm^−1^) correspond to the LO-TO pair of a 6-ring network. These assignments are consistent with the report that the discrepancy of LO-TO splitting for 6-ring silica locates within the wavenumber boundary of 140 to 160 cm^−1^ 28,29. The proportion of LO mode is reported to be an indicator showing the extent of condensation. Primeau *et al.* demonstrated that the LO_3_ vibration mode is not observable if the sol-gel process does not sufficiently evolve and the siloxane groups are not fully converted^30^. In our work, the LO_3_ mode at ~1205 cm^−1^ is detectable after 6 h as shown in Fig. 3B, indicating the high degree of siloxane condensation.

**Figure 6:**
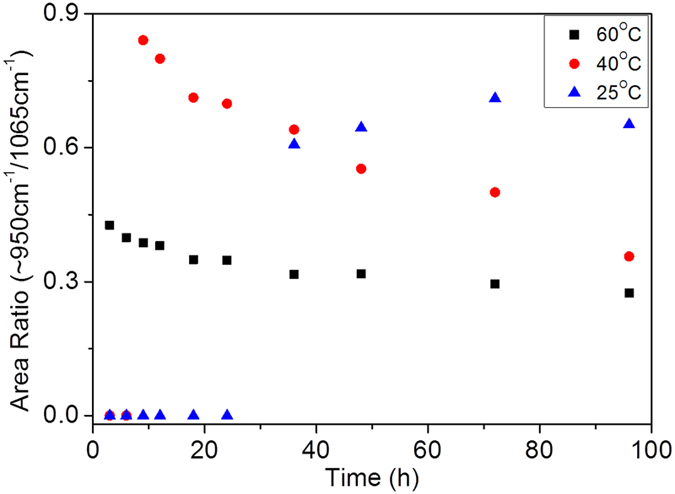
Comparative FTIR ratios of Si-OH to Si-O-Si as a function of time at different reaction temperatures (25, 40 and 60 °C) (estimated standard error = ±4%).

The ratio of the peak area originated from comparing the area of uncondensed silicon species (peaks VI and VII) to that from condensed silica (the dominant siloxane band IV). This can be used as an indicator of the degree of hydrolysis and condensation reactions. Figure 6 shows the calculated peak area ratio as a function of reaction time at different temperatures. It can be observed from Fig. 6 that the peak area ratio of Si-O(H)/Si-O-Si for all the samples increases and then decreases at different point in the gelling time. On a closer examination, the induction period of hydrolysis is significantly delayed with decreasing temperature from 6 to 25 h for the 40 and 25 °C samples, respectively. As such, the 25 and 40 °C samples had not completely reached equilibrium at the end of 96 h. This result was expected for two reasons. Firstly, sol-gel reactions are known to be promoted by temperature. Secondly, due to solvent evaporation, the sol-gel species are forced to come in close proximity which promotes chemical reactions.

More importantly, as shown in Fig. 6, the point when the Si-OH/Si-O-Si ratio reaches a maximum were measured at 3, 9 and 72 h for 60, 40 and 25 °C, respectively. In other words, this time indicates that hydrolysis is being overtaken by condensation reactions, and thus more siloxane species are being generated at the expense of the silanol species regardless of their molar absorptivities. This further indicates that the shift in the reaction equilibrium is favoured towards the condensation reactions. These results demonstrate that the sol-gel process in phase-separated system is suppressed by low temperature due to a slower hydrolysis reaction and subsequently inhibits the condensation reactions. This is not so dissimilar to those reported for homogeneous TEOS derived silica sol-gel systems^14^,^27^,^31^.

**Figure 7:**
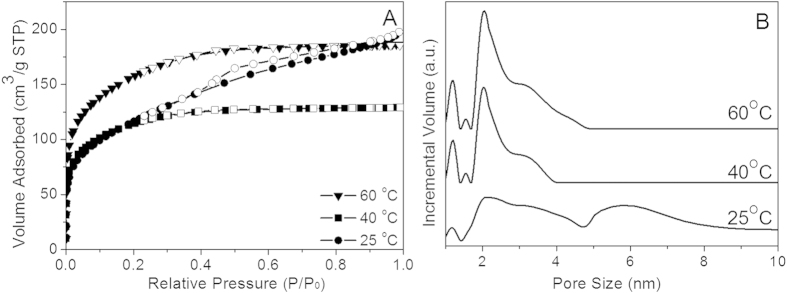
(**A**) N_2_ adsorption (solid symbols) and desorption (open symbols) isotherms and (**B**) DFT pore size distribution of silica xerogels dried at 25, 40 and 60 °C.

The structure properties of the resultant xerogels after calcination were studied by N_2_ sorption. It can be observed from Fig. 7A that xerogels dried at 40 and 60 °C show Type I isotherms, indicating that micropores are dominant in their texture. The xerogel dried at 60 °C had an adsorption isotherm which achieved saturation at a higher relative pressure than the sample dried at 40 °C, in addition to a larger amount of N_2_ volume adsorbed. For the xerogel dried at 25 °C, the N_2_ adsorption and desorption isotherms exhibit a type IV hysteresis loop, characteristic of a mesoporous structure. In addition, the DFT pore size distribution (Fig. 7B) of the 25 °C xerogel exhibited a much wider profile than the others, also implying a larger pore size. These results illustrate that when the drying temperature decreases from 60 to 40 °C, the effect on structure properties is not obvious and the product is still microporous. Whereas when the temperature is further decreased to 25 °C, a mesoporous material is obtained.

**Figure 8:**
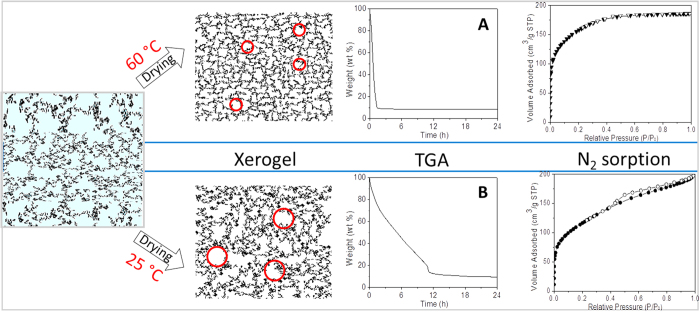
TGA curves of silica sol-gel solutions and schematic silica matrix obtained after dying at 25 and 60 °C. Red circles are used to illustrate pores.

To shed further light on the structural formation of the xerogels as a function of the drying temperature, TGA was conducted by holding the sample at constant temperatures until completely dried. It can be observed from Fig. 8A that, for the gels dried at 60 °C, a sharp weight loss appears after 2 h. This was much faster than for the sample dried at 25 °C, as shown in Fig. 8B. The system keep losing weight due to evaporation of solvent and by-product of sol-gel reactions until equilibration is achieved after 4 h at 60 °C or 20 h at 25 °C. The equilibration of the sol-gel process at 60 °C appeared at much earlier period than the 25 °C system, which is consistent with the FTIR results in Fig. 6.

Figure 8 also shows a schematic of the ES40 sol-gel process. The mesoporous structure obtained at 25 °C in principle could be attributed to the much slower solvent evaporation during drying as confirmed by the TGA curves. It has been reported that the degree of shrinkage of silica network during drying depends on the relative rate of solvent evaporation and stiffening of the network^4^, as it is the case for TEOS derived sol-gel. As solvent evaporates, surface tension and drying stress created in the gel results in the collapse of the network^32^. Concomitantly, the stiffness of network also enhanced due to increasing crosslinking of the silica chains by sol-gel reaction and decreasing porosity through shrinking. This in turn acts against network shrinkage. In the case of the ES40 derived sol-gel, it could be expected that the gel dried at 25 °C would have a denser structure due to the higher content of Si-OH groups as shown in Fig. 6. These groups are generally deemed to collapse under capillary forces, whilst Si-O-Si bridges stiffen the silica structure and oppose densification. This is not the case for the ES40 derived sol-gel process, suggesting that a different mechanism is occurring.

ES40 is a partially condensed TEOS, so it is no longer a monomer leading to oligomers like TEOS. The mesoporosity of the gel dried at 25 °C suggests that the slow reaction process led to the formation of unreacted ES40 cages filled with solvent. These cages were large enough to have lower capillary pressures and reduced drying stress. These cages hindered the ES40 oligomers to get in closer contact to react, and favoured the formation of mesoporous structures. This can be clearly observed in Fig. 7B, as the pore size distribution for the gel dried at 25 °C broadened from ~5 to 9 nm, which is not observed for the gels dried at 40 and 60 °C. In the case of the latter, faster evaporation allowed for closer contact of the ES40 oligomers and fast reaction leading to the formation of Si-O-Si bridges and structural interpenetration as evidenced by the microporous character of their adsorption isotherms. Hence, these results strongly suggest that solvent evaporation played an important role in the structural formation of ES40 derived sol-gel, coupled with the effect of reaction.

**Figure 9:**
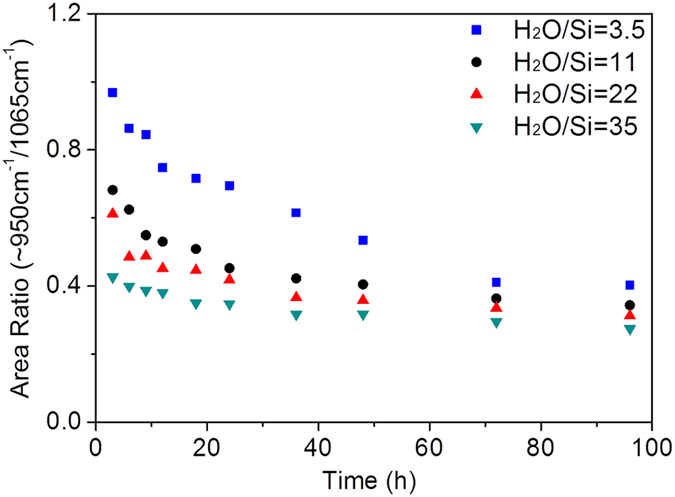
Comparative FTIR ratios of Si-O(H) to Si-O-Si as a function of time with different H_2_O/Si ratios (3.5, 11, 22 and 35) (estimated standard error = ±4%).

The effect of H_2_O/Si ratios on phase-separated sol-gel performance was also studied as water is widely-recognized as an important hydrolysing agent in sol-gel reaction, whilst keeping nitric acid constant. Based on the FTIR results (Fig. 6), for a molar ratio of H_2_O/Si = 35, the sol-gel process was promoted by increasing the reaction temperature during the drying process at 60 °C. Therefore, samples with reduced H_2_O/Si ratio of 22, 11 and 3.5 were prepared at the same temperature for comparison. A homogeneous solution was obtained when H_2_O/Si ratio decreased to 3.5, as per Fig. 1. The Si-O(H)/Si-O-Si ratio in Fig. 9 increased at the beginning of the reaction and decreased afterwards until equilibrium was reached. The Si-O(H)/Si-O-Si ratio during the whole drying process decreased with increasing H_2_O/Si ratio. It is also noteworthy that the Si-O(H)/Si-O-Si ratio of the homogeneous solution (H_2_O/Si = 3.5) was significantly higher than the phase-separated systems. This can be attributed to the dilutive effect of the solvent on the homogeneous sol-gel reaction solution, which suppresses the condensation reaction. On the contrary, in phase-separated systems, the reactive species are highly concentrated around the reaction interface thus promoting further condensation. The time point when equilibrium was reached was also affected by the water content, which increased from 36 h to 72 h when H_2_O/Si ratio decreased from 35 to 3.5. These results demonstrate that condensation is furthered by a high H_2_O/Si ratio. This is expected as water is a hydrolysing agent commonly employed in the sol-gel process, which promotes the condensation reaction, and yields a lower Si-O(H)/Si-O-Si ratio and faster equilibrium.

By the same token, HNO_3_ promotes the sol-gel process due to its catalytic effect. By keeping the acid concentration constant, all of the initial sol systems had a measured pH value of around 1 in the top phase. When the pH is lower than the point of zero charge (pH ~ 2) of polymeric silica species, the sol-gel reaction at the interface proceeds through the protonated alkoxide group (SiORH^+^) during the hydrolysis and this makes the silicon atom more electrophilic and more reactive towards water. Once the silanol group is formed as a result of hydrolysis, the silanol species is expected to preferentially migrate into the aqueous phase (top phase). This is further promoted by the acid to form the protonated silanol groups (SiOH_2_^+^) during the condensation reactions. Since the acid concentration was kept constant, the promotion of hydrolysis and subsequent condensation reactions would be aided by higher water concentration which ultimately yields a lower Si-O(H)/Si-O-Si ratio.

**Figure 10:**
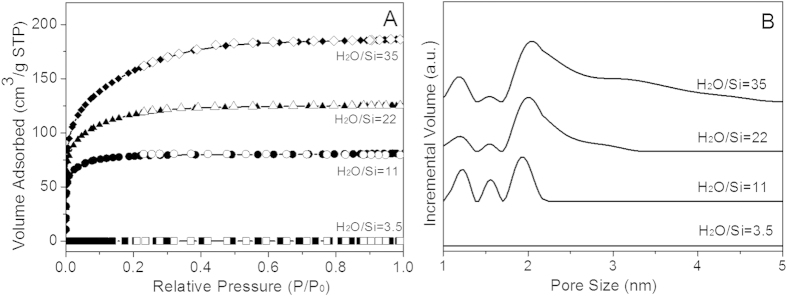
(**A**) N_2_ adsorption (solid symbols) and desorption (open symbols) isotherms and (**B**) DFT pore size distribution of silica xerogels prepared with different H_2_O/Si ratios (35, 22, 11 and 3.5).

The N_2_ sorption results in Fig. 10A shows that xerogel prepared from homogeneous sol-gel solution with low H_2_O ratio of 3.5 was mainly dense with no N_2_ adsorbed. The other isotherms are all Type I, indicating microporous networks. The volume adsorbed increases with increasing H_2_O ratios, implying a larger total pore volume. This was confirmed through DFT pore size distributions (Fig. 10B) which shows an increasing fraction of larger pores with increasing H_2_O ratio. Similar to the homogeneous reaction system, such an effect can be explained by acceleration of hydrolysis resulting from a high H_2_O ratio. This is also indicated by FTIR analysis in Fig. 9. As more reactive sites appear, silica chains with higher degree of crosslinking are produced. These are beneficial to the formation of larger silica particles and a more open silica network with a lower silanol to siloxane ratio^19^.

**Figure 11:**
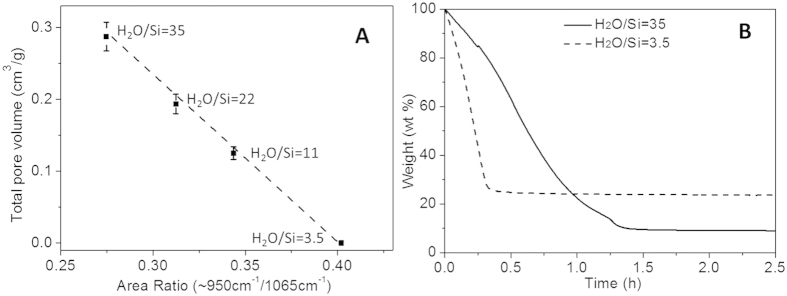
(**A**) Total pore volume of silica xerogels as function of comparative FTIR area ratios of silanol to siloxane vibrational peaks (**B**) TGA curves of sol-gel solutions drying at 60 °C.

The relationship between total pore volume of silica xerogels and comparative silanol to siloxane ratios is shown in Fig. 11A. Based on these results, it can be deduced that high porosity can be obtained through phase-separated sol-gel systems with high H_2_O to Si ratios. Further the textural properties of the xerogels prepared from phase-separated solution can also be easily tuned by changing reactant ratios. Figure 11B shows that the solvent evaporation in the homogenous solution is much faster than that in the phase-separated solution with high H_2_O/Si ratio. Faster evaporation led to larger drying stresses which caused more network compression. As the homogeneous sample had a higher content of silanol groups, indicating a limited crosslinking of the silica chains, this weak structure was unable to oppose the drying stress. Hence the silica network collapsed, resulting in a dense or perhaps ultra-microporous structure. Contrary to this trend, the slower evaporation rate and higher siloxane content induced stiffening of the network and prevented shrinking of the phase-separated gels.

## Conclusions

The heterogeneous sol-gel system of ES40 in acidified water-ethanol co-solvent successfully produced silica microstructure with tailored microporosity despite a reduced miscibility and prolonged phase separation, which is contrary to that of the conventional homogenous systems commonly reported (eg with TEOS as the precursor). The evolution of the phase-separated sol-gel process demonstrated that the ES40 sol-gel reactions can occur, commencing at the interface of the two layers; namely ES40 (bottom) and co-solvent (top) phases. This may be followed by migration of the silanol groups into the co-solvent phase generated by hydrolysis leading to the formation of the siloxane groups by condensation. These conclusions are supported by the temporal dependence of FTIR area ratio of Si-O(H)/Si-O-Si, which increased at the beginning of the sol-gel process in the top phase due to the hydrolysis reaction and then decreased over time due to condensation reactions.

The phase-separated ES40 sol-gel process was also found to be affected by both the reaction temperature and the initial H_2_O/Si ratios. Higher temperatures favoured faster solvent evaporation of the top phase thus bringing the reactive species in closer proximity to facilitate their reactions. Microporous xerogels were produced at 60 and 40 °C, while mesoporous structures were obtained when the reaction temperature was carried out at 25 °C. The formation of larger pores under lower temperature condition was attributed to two factors. Firstly, slow solvent evaporation reduced the drying stress and consequently the silica network compression was weak. Secondly, the oligomer silica precursor ES40 can form cages, inducing lower capillary forces and larger pores.

The textural properties of the xerogels can also be easily tuned by changing the reactant ratios, as water is an important hydrolyzing agent but its immiscibility also promotes phase separation. By increasing the H_2_O/Si ratio, the phase-separated sol-gel process dried much slower than conventional homogeneous sols, which in turn promoted an accelerated hydrolysis at the interface. This yielded highly condensed porous silica xerogels, contrary to a densified silica microstructure derived from homogeneous systems with a lower H_2_O/Si ratio and faster evaporation process. In phase-separated sol-gel systems, most reactive species are concentrated around the interface, and this close proximity facilitated further condensation reactions and crosslinking of silica chains. In turn, this induced stiffening of the network making it more resistant to compression and densification.

## Method

ES40 was purchased from Colcoat Co., Japan. Ethanol (EtOH, AR grade) and all the chemicals were used as received. Initially, distilled reverse osmosis (RO) water was mixed with ethanol and 1 M nitric acid (HNO_3_) to obtain solution with pH ~ 1 with a fixed H_2_O/HNO_3_ ratio of 110. Then ES40 was added to the mixture dropwise under stirring in an ice bath and the molar ratio of the reactants Si (ES40): H_2_O: EtOH was kept at 4: 140: 15, hence for this sample, H_2_O/Si is 35. After stirring for 10 mins, the solution was kept undisturbed in an open Schott bottle and dried in oven at varying temperatures of 25, 40 or 60 °C for 96 h. As soon as stirring ceased, the mixture developed into two phases. A series of samples prepared from different molar ratios of H_2_O to Si (35, 22, 11 and 3.5) were also studied.

Fourier transform infrared (FTIR) spectroscopy characterization was performed with a Shimadzu IRAffinity-1 with a Pike MIRacle diamond attenuated total reflectance (ATR) attachment. The analysis was performed over a wavenumber range of 4000–600 cm^−1^. Background subtraction and peak deconvolution of the spectra were performed using the Fityk program. Samples were analyzed in both the sol-gel and xerogel states. The evolution of the sample was investigated by characterising 20 μL aliquot using micropipette taken from each of the liquid phase and xerogel powder during drying times. The contribution of ethanol solvent in the sol-gel samples was removed from the FTIR spectra by normalizing against the pure ethanol spectrum using the characteristic peak ~870 cm^−1^ for the ethanol and then followed by background subtraction^13^. To cross-check this procedure, no absorption peaks between 3000 and 2800 cm^−1^ range attributable to the ethoxy groups of ethanol or ES40 precursor were observed after the background subtraction. This means that the contribution of ethoxy groups of the ES40 precursor between 1300 and 900 cm^−1^ vibrational range is also negligible and that the spectra contained only the silica species. After this step, peak fitting analysis between 1300 and 900 cm^−1^ spectral range was performed for all the spectra by using a local baseline and Gaussian peaks with the square of correlation coefficient values ≥0.95. The same number of peaks was used in all the spectral peak fitting. The half width at half maximum (HWHM) was fixed for each peak, while the peak position was allowed to change slightly to realize qualified fitting. The gravimetric analyses of the silica sol-gel solutions were performed on a Shimadzu thermogravimetric analyzer TGA-50 using air flow rate of 5 mL min^−1^ at room temperature, 40 and 60 °C until mass loss was no longer detected. Nitrogen physi-sorption analysis was conducted at −196 °C on a Micromeritics TriStar 3020 apparatus. Samples were degassed at 200 °C overnight before each measurement. The pore size distributions were determined from adsorption branch of the isotherms using the Density Functional Theory (DFT) model of cylindrical pores with oxide surfaces.

## Additional Information

**How to cite this article**: Wang, S. *et al.* Ternary Phase-Separation Investigation of Sol-Gel Derived Silica from Ethyl Silicate 40. *Sci. Rep.* **5**, 14560; doi: 10.1038/srep14560 (2015).

## Supplementary Material

Supplementary Figures

## Figures and Tables

**Table 1:** Band assignments of the FTIR vibrations of the reactants in Fig. 2^20^,^21^,^22^,^23^.

Wavenumber (cm^−1^)	Vibration mode	Chemicals
~3320	𝜈(O-H)	water, ethanol, Si-OH
~3000–2800	𝜈(C-H)	ethanol, Si-OCH_2_CH_3_
~1640	δ(H-O-H)	water
~1168	ρ(CH_3_)	Si-OCH_2_CH_3_
~1101, 1061	𝜈_as_ (C-O)	Si-OCH_2_CH_3_
~1086	𝜈(C-C)/(C-O)	ethanol
~1045	ρ(CH_3_/CH_2_)	ethanol
~965	ρ(CH_3_)	Si-OCH_2_CH_3_
~878	𝜈(C-C)/(C-O)	ethanol
~790	𝜈(C-O)	Si-OCH_2_CH_3_

**Table 2:** FTIR band assignments of the deconvoluted peaks of the xerogels in Fig. 5^28^,^33^.

Deconvoluted peaks	Wavenumber (cm^−1^)	Vibration mode	Chemicals
I	~1205	LO_3_ mode of 𝜈_as_(Si-O-Si)	6-ring siloxane (SiO)_6_
II	~1146	LO_4_ mode of 𝜈_as_(Si-O-Si)	4-ring siloxane (SiO)_4_
III	~1105	TO_4_ mode of 𝜈_as_(Si-O-Si)	4-ring siloxane (SiO)_4_
IV	~1065	TO_3_ mode of 𝜈_as_(Si-O-Si)	6-ring siloxane (SiO)_6_
V	~1035	𝜈_as_(Si-O-Si)	chain silicate
VI	~962	𝜈(Si-OH)	silanol
VII	~934	𝜈(Si-O^−^)	silica open rings
