# Supplementary material for: Ternary Phase-Separation Investigation of Sol-Gel Derived Silica from Ethyl Silicate 40
Source: Sci Rep. 2015 Sep 28;5:14560. doi: 10.1038/srep14560 (PMC4585945; doi:10.1038/srep14560)
Supplement: Supplementary Figures [file srep14560-s1.pdf]

Ternary Phase-Separation Investigation of Sol-Gel Derived Silica from Ethyl Silicate 40

Shengnan Wang, David K. Wang*, Simon Smart, João C. Diniz da Costa

Supplementary Information

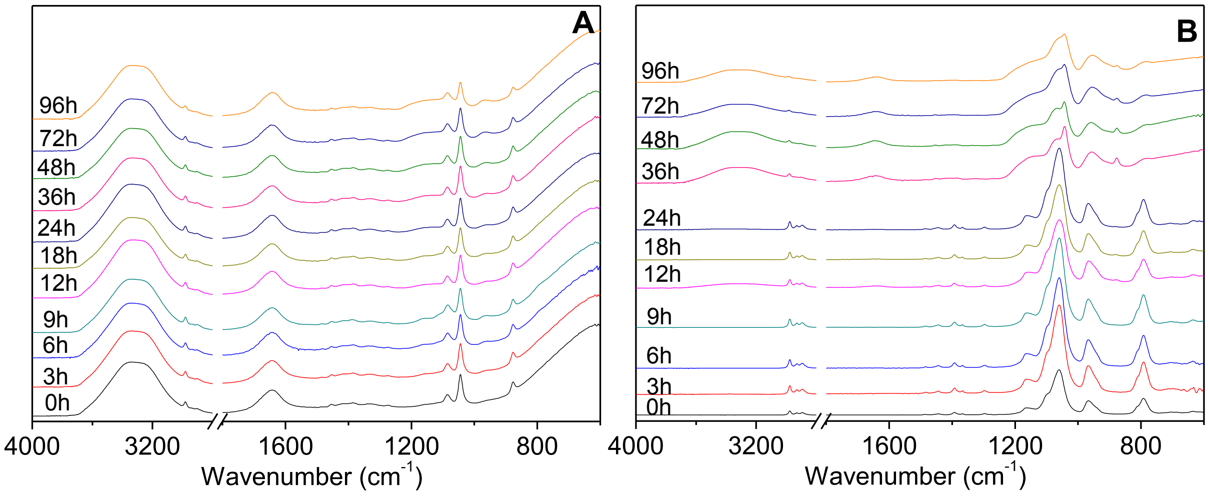

Figure S1. FTIR spectra of top (A) and bottom (B) phase in ES40 sol-gel solutions at 25 °C.

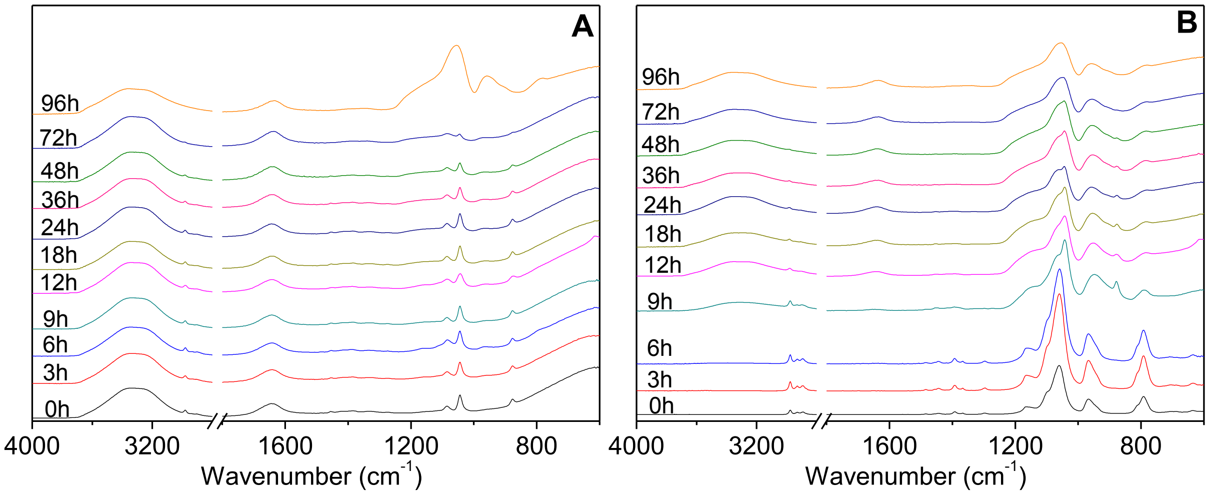

Figure S2. FTIR spectra of top (A) and bottom (B) phase in ES40 sol-gel solutions at 40 °C.
